# Loss-of-Function Variants in the *SYNPO2L* Gene Are Associated With Atrial Fibrillation

**DOI:** 10.3389/fcvm.2021.650667

**Published:** 2021-03-09

**Authors:** Alexander Guldmann Clausen, Oliver Bundgaard Vad, Julie Husted Andersen, Morten Salling Olesen

**Affiliations:** ^1^Department of Biomedical Sciences, Faculty of Health and Medical Sciences, University of Copenhagen, Copenhagen, Denmark; ^2^Laboratory for Molecular Cardiology, Department of Cardiology, The Heart Centre, Rigshospitalet, University Hospital of Copenhagen, Copenhagen, Denmark

**Keywords:** atrial fibrillation, genetics, arrhythmia, splice site variant, cardiomyopathy, cardiology

## Abstract

Multiple genome-wide association studies (GWAS) have identified numerous loci associated with atrial fibrillation (AF). However, the genes driving these associations and how they contribute to the AF pathogenesis remains poorly understood. To identify genes likely to be driving the observed association, we searched the FinnGen study consisting of 12,859 AF cases and 73,341 controls for rare genetic variants predicted to cause loss-of-function. A specific splice site variant was found in the *SYNPO2L* gene, located in an AF associated locus on chromosome 10. This variant was associated with an increased risk of AF with a relatively high odds ratio of 3.5 (*p* = 9.9 × 10^−8^). *SYNPO2L* is an important gene involved in the structural development and function of the cardiac myocyte and our findings thus support the recent suggestions that AF can present as atrial cardiomyopathy.

## Introduction

Atrial Fibrillation (AF) is the most common cardiac arrhythmia with more than 33 million people affected worldwide (1). The disease has been associated with an increased risk of stroke, heart failure as well as increased all-cause mortality (2). While AF is a complex disease associated with multiple risk factors including age, sex, lifestyle and existing heart disease, a significant genetic component of the disease has also been established (3). In 2018, a meta-analysis of genome wide association studies (GWAS) of AF identified a total of 134 loci associated with AF (4). Many of these loci have been linked to genes related to ion channels, structural proteins of the myocardium and transcription factors (5). However, a large number of the identified loci are located in non-coding regions and in the vicinity of multiple genes. More work is needed to identify the causal genes mediating the observed associations. The purpose of this paper is to investigate potentially causal genes at known GWAS loci in order to further elucidate the mechanisms behind AF pathogenesis. This was done by searching genes in a publicly available dataset containing phenotypes linked with imputed genotypes for AF-associated variants in loci previously associated with AF in GWAS.

## Materials and Methods

To identify genes likely to be driving the association between the observed GWAS loci and AF, we searched the FinnGen database (6) for variants significantly enriched in AF cases. Only variants located in one of the 134 GWAS loci previously identified in our GWAS meta-analysis (4) were selected for further investigation. To increase the likelihood of the identified variants contributing to the pathogenesis of AF development, we confined our analysis of the FinnGen data to only include predicted loss-of-functions variants. These were defined as variants leading to a pre-mature stop codon, deletions or insertions leading to a frameshift or variants involving splice sites. To further evaluate the identified candidate genes, we analyzed mRNA expression in right atrial appendage tissue integrated with genotype data obtained through the Genotype-Tissue Expression (GTEx) project. This was done by searching the publicly available GTEx release v8 database (https://gtexportal.org/home/gene/SYNPO2L). A brief description of the used data from the FinnGen database and the GTEx project is given below.

### FinnGen Database

The publicly available FinnGen database contains genotype data from Finnish biobanks linked with corresponding national health registry data from 12,859 AF cases and 73,341 controls. Data from the FinnGen database was retrieved from freeze number 3. FinnGen participants were genotyped by the FinnGen investigators using various Illumina and Affymetrix GWAS arrays. This was followed by a genotype imputation by the Beagle v.4.1 software using 3,775 high coverage whole genome sequences of Finnish origin as a population specific reference panel (6). We used the FinnGen disease endpoint “Atrial Fibrillation and Flutter.” The SAIGE software was used for the genetic association tests of the FinnGen data. SAIGE is a mixed model logistic regression R/C++ package that is able to adjust for relatedness of the analyzed samples while also controlling for unbalanced case-control ratios that can otherwise lead to inflated type 1 error rates (7). SAIGE first generates relatedness estimates and a null model. Next, the null model is fitted with the respective SNP, and the genetic kinship matrix and the model itself is then adjusted for the following covariates: sex, age, 10 principal components and genotyping batch. For more information on the included biobanks, disease endpoint definitions and genotype data in the FinnGen database, please visit https://finngen.gitbook.io/documentation/v/r3/.

### GTEx Project

The GTEx Project database contains analyses of mRNA levels in 47 different tissues, including atrial appendage tissue obtained from 429 deceased donors with available genotype data. The data was obtained by massive parallel sequencing of RNA strands using Illumina TrueSeq RNA sequencing followed by qPCR (8).

## Colocalization Analysis

In order to further explore the genes responsible for the identified genome-wide associated risk locus, we performed a colocalization analysis. Using the LocusFocus tool (9), we tested whether the GWAS signals are colocalized with eQTL signals, and therefore are likely to share a causal variant. The LocusFocus tool utilizes the *Simple Sum* colocalization method based on a frequentist framework. To perform the colocalization analysis, we used the publicly available FinnGen AF GWAS summary statistics and integrated them with cis-eQTL data from atrial appendage tissue from the GTEx project v8. For an identified locus, we used GWAS summary statistics from all SNPs within a 100 kb window centered around the identified lead SNP. The performance of the Simple Sum colocalization method compared with other colocalization methods has been described by Gong et al. (10).

## Pathway Analysis

We performed an *in-sillico* pathway analysis of interactions between the protein product of *SYNPO2L* and other proteins in AF, using the tool String (v11) (11). String is a large database of protein-protein interactions, derived from multiple sources, including both physical and functional associations. We conducted a search of proteins thought to be important in structural function of cardiomyocytes, as defined in a recent review of AF genetics by Andersen et al. (12). Afterwards a search of all proteins was done, limited to the 10 interactions predicted with highest confidence. Evaluation of confidence in interactions have been described in detail by Szklarczyk et al. (11).

## Evaluation of Variant Deleteriousness

Variants in candidate genes were evaluated using the *in silico* tool Combined Annotation Dependent Depletion Score (CADD), in order to predict the effects and deleteriousness of the concerned variants (13). This method uses a machine learning model to incorporate multiple different forms of variant annotations into one integrated score, in order to better identify causal variants. Variants with a CADD-score >20 are predicted to be among the 1% most deleterious variants in the human genome. Those with a CADD-score >30 are predicted to be among the 0.1% most deleterious.

## Results

### FinnGen Database

A total of 119 variants were associated with AF in the FinnGen study. Of these, 13 variants were located in genes in loci previously identified in our GWAS meta-analysis. The 13 variants are presented in Table 1. Of the 13 variants, only one variant, rs766868752, was predicted to be a loss-of-function variant. This variant was located in the *SYNPO2L* gene. The variant was imputed with an INFO-score of 0.972, indicating a very high confidence imputation.

**Table 1:** Variants associated with AF in the GWAS meta-analysis by Roselli et al. that were located near genes also associated with AF in the FinnGen study.

**Meta-analysis SNP**	**Chr**	**Nearest gene**	**RR (95 % CI)**	***p*-value**	**Effect/ref allele**	**Effect allele frequency**	**SNP placement relative to nearest gene**	**FinnGen variant rsid**	**FinnGen variant OR (95% CI)**	***p*-value**	**Effect/ref allele**	**Effect allele frequency**	**FinnGen variant placement relative to nearest gene**
rs880315	1	*CASZ1*	1.04 (1.03–1.06)	*p* = 5.0 × 10^−9^	C/T	0.37	Intronic	rs34071855	1.1 (1.07–1.15)	*p* = 3.6 × 10^−8^	G/C	0.41	Intronic
rs12044963	1	*KCND3*	1.08 (1.06–1.11)	*p* = 1.6 × 10^−12^	T/G	0.18	Intronic	rs34119223	0.88 (0.84–0.92)	*p* = 1.3 × 10^−7^	T/TCC	0.19	Intronic
rs35504893	2	*TTN*	1.09 (1.08–1.11)	*p* = 6.9 × 10^−25^	T/C	0.25	Intronic	rs16866400	1.1 (1.09–1.19)	*p* = 4.4 × 10^−9^	A/G	0.22	Intronic
rs6810325	3	*CAND2*	1.08 (1.06–1.09)	*p* = 5.2 × 10^−23^	C/T	0.63	Intronic	rs7650482	1.1 (1.06–1.14)	*p* = 1.3 × 10^−6^	G/A	0.61	Intronic
rs7632427	3	*EPHA3*	1.04 (1.03–1.06)	*p* = 1.10 × 10^−8^	T/C	0.61	Downstream	rs7633500	0.92 (0.88–0.95)	*p* = 4.2 × 10^−6^	A/G	0.45	Intronic
rs73366713	6	*ATXN1*	1.11 (1.09–1.14)	*p* = 5.8 × 10^−21^	G/A	0.86	Intronic	rs112583508	0.86 (0.81–0.92)	*p* = 4.7 × 10^−6^	A/G	0.098	Intronic
rs3176326	6	*CDKN1A*	1.06 (1.04–1.08)	*p* = 8.0 × 10^−11^	G/A	0.80	Intronic	rs3176323	0.9 (0.86–0.94)	*p* = 3.0 × 10^−7^	C/T	0.29	Intronic
rs17079881	6	*SLC35F1*	1.09 (1.07–1.11)	*p* = 4.2 × 10^−16^	G/A	0.14	Intronic	rs111862156	1.5 (1.28–1.80)	*p* = 1.7 × 10^−6^	T/C	0.012	Intronic
rs11773845	7	*CAV1*	1.12 (1.11–1.14)	*p* = 4.6 × 10^−58^	A/C	0.59	Intronic	rs3807990	0.89 (0.86–0.93)	*p* = 5.5 × 10^−8^	T/C	0.28	Intronic
rs60212594	10	*SYNPO2L*	1.12 (1.09–1.14)	*p* = 6.5 × 10^−27^	G/C	0.85	Intronic	rs766868752	3.5 (2.20–5.48)	*p* = 9.9 × 10^−8^	A/C	1.3·10^−3^	Splice site donor
rs113819537	12	*SSPN*	1.05 (1.03–1.07)	*p* = 2.2 × 10^−09^	C/G	0.74	Upstream	rs113819537	0.9 (0.87–0.94)	*p* = 4.3 × 10^−7^	G/C	0.31	5′ UTR
rs883079	12	*TBX5*	1.13 (1.11–1.14)	*p* = 1.3 × 10^−51^	T/C	0.69	3′ UTR	rs7312625	1.1 (1.07–1.16)	*p* = 1.9 × 10^−7^	A/G	0.7	Intronic
rs2359171	16	*ZFHX3*	1.21 (1.19–1.23)	*p* = 2.9 × 10^−100^	A/T	0.19	Intronic	rs2359171	1.2 (1.14–1.24)	*p* = 3.8 × 10^−15^	A/T	0.23	Intronic

*All FinnGen variants had an imputation info score >0.95. RR, Relative Risk; OR, Odds Ratio; CI, Confidence Interval*.

The rs766868752 variant in *SYNPO2L* is characterized as a splice site donor variant and is located on position 73,655,817 on the first base of intron 1. In carriers of the variant, the C base of the *non-template strand* is replaced with an A base. Thus, the normal splice donor site of 5′ GT 3′ in the template strand is changed to a 5′ TT 3 ′. In the 12,859 AF cases in the FinnGen study, the variant was found with a minor allele frequency (MAF) of 0.002585. In the 73,341 control participants, the variant was found with a MAF of 0.001022. We found an increased Odds Ratio (OR) of developing AF of 3.5 in persons carrying the c.105+1G>T variant in *SYNPO2L* (*p* = 9.9 × 10^−8^). According to the gnomAD browser, a database containing more than 76,000 exome sequences, the specific variant was almost exclusively found in people of Finnish descent (14). Of the 41 carriers of the mutation in the gnomAD database, 40 was of Finnish descent and only one carrier was of non-Finnish European descent. Only one other splice site donor variant has been identified in the gnomAD v3 database, suggesting that splice site mutations in *SYNPO2L* are rare and likely deleterious.

**Figure 1:**
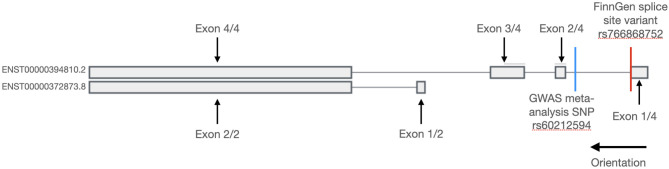
Transcripts of the *SYNPO2L* gene. Exons are shown with thick bars and introns are shown with thin lines. The *SYNPO2L* gene is expressed in a short and a long isoform. The splice site variant found in the FinnGen database is shown with a red line and is located in the first intron of the long isoform. The position of the common SNP, rs60212594, identified in the GWAS meta-analysis is shown with a blue line.

Two isoforms of the *SYNPO2L* genes are expressed *in-vivo*. A long isoform with the ID ENST00000394810.2 as well as a short isoform with the ID ENST00000372873.8 (14). The splice site mutation found in the FinnGen study is located in the long transcript as seen in Figure 1. As the only gene positioned in a locus identified in our GWAS meta-analysis that also was found to have predicted loss-of-function variants enriched in the FinnGen study population, the *SYNPO2L* gene was selected for further investigation.

### Colocalization Analysis

**Figure 2:**
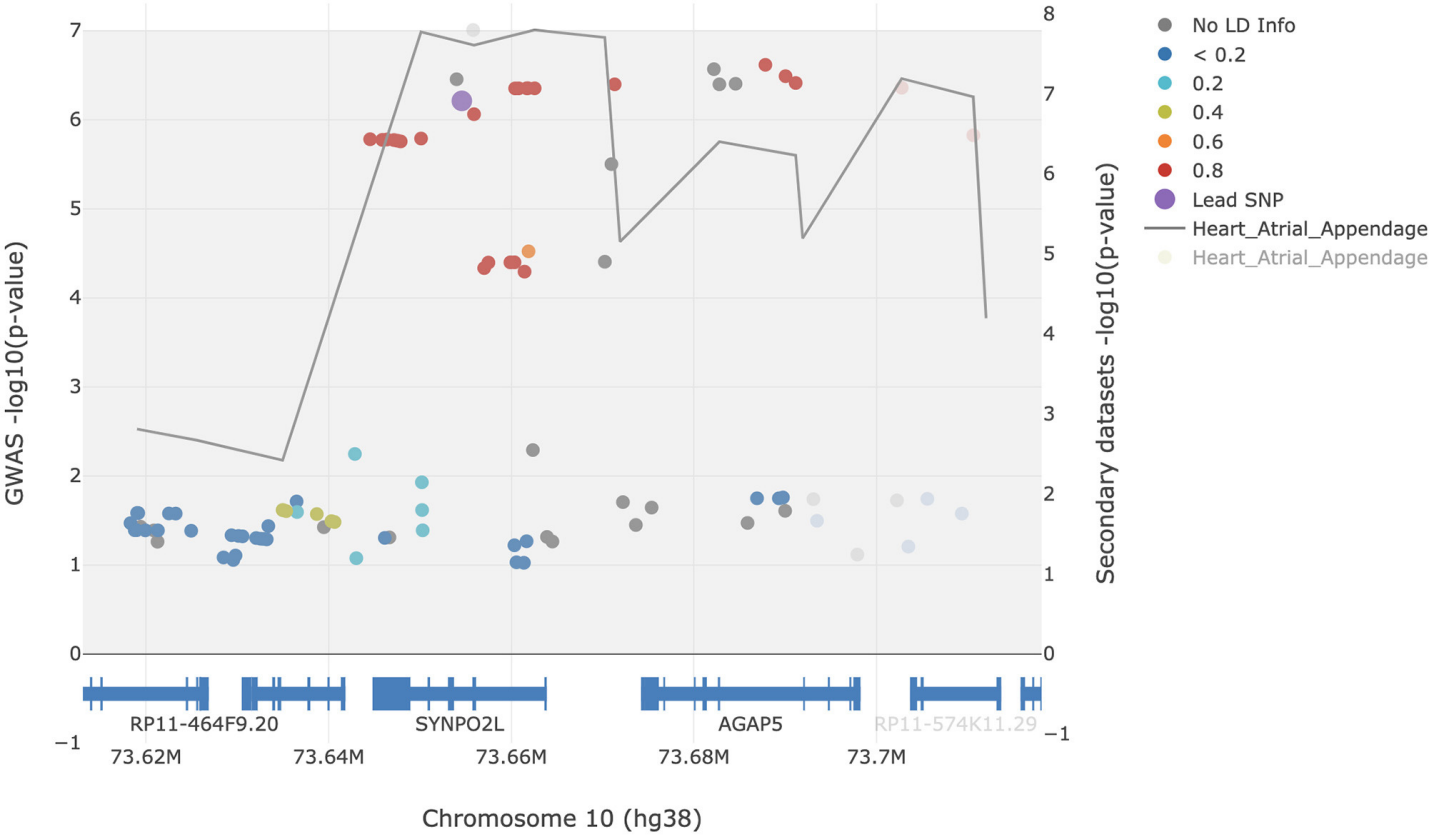
Colocalization analysis of AF GWAS signals with eQTL signals of *SYNPO2L* in atrial appendage tissue. Filled circles represent AF GWAS -log_10_(p-values) (left y-axis). The gray line represents the eQTL signals and traces the lowest p-value [right y-axis, showing -log_10_(*p*-values)] per window of 22.5 bp. The rs60212594 SNP was defined as the lead SNP and is presented in purple. The European 1000 Genomes Project was used for the LD calculations in reference to the lead SNP. The figure was generated using the LocusFocus tool.

To determine whether the GWAS signals at the *SYNPO2L* locus are likely to be driven by changes in *SYNPO2L* expression in atrial appendage tissue, we performed a colocalization analysis using the LocusFocus tool. Our analysis supports a strong colocalization of GWAS signals with eQTLs of *SYNPO2L* in atrial appendage tissue with a *Simple Sum p*-value of 2.1 × 10^−6^. The colocalization analysis has been visualized in Figure 2.

### GTEx Project Results

**Figure 3:**
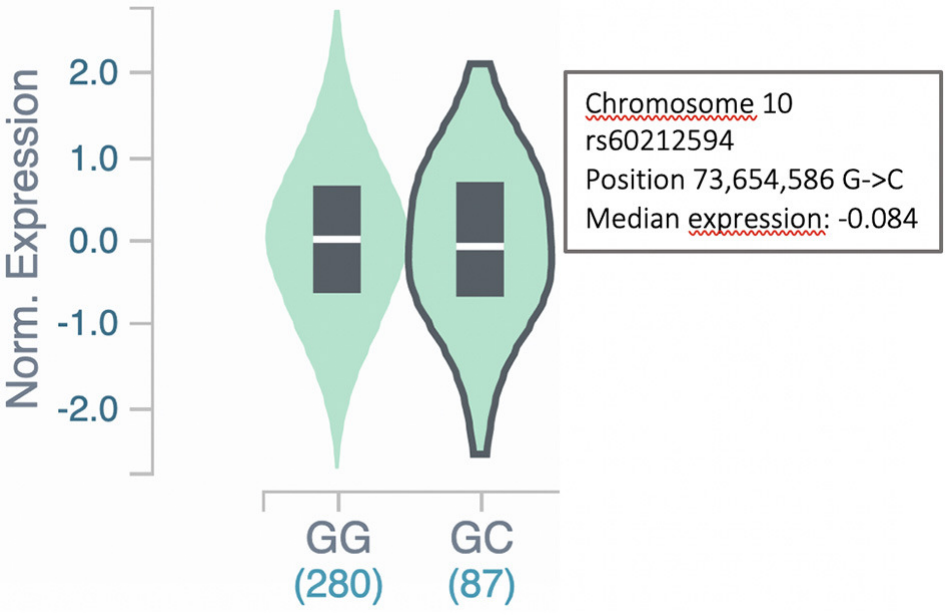
*SYNPO2L* expression in carriers of the rs60212594 SNP. Expression of the *SYNPO2L* gene is decreased (*p* = 2.9 × 10^−8^) in heterozygous carriers of the C allele of the rs60212594 SNP associated with AF. The data and figure were obtained from the GTEx project eQTL analysis (https://gtexportal.org/home/snp/rs60212594).

To further assess the involvement of the *SYNPO2L* gene in AF, we examined whether the expression of the *SYNPO2L* gene was affected in carriers of the SNP rs60212594. This common SNP is located at the *SYNPO2L* locus and was associated with an increased risk of AF in our GWAS meta-analysis (4). This was done by a quantitative trait locus (cis-eQTL) analysis using data from the GTEx project. The rs60212594 SNP identified in the GWAS was shown to be associated with a minor but significant change in *SYNPO2L* expression (15). Carriers of the C risk allele had a 0.08-fold decrease in *SYNPO2L* expression with a *p*-value of 2.9 × 10^−8^, which is illustrated in Figure 3.

### Pathway Analysis

**Figure 4:**
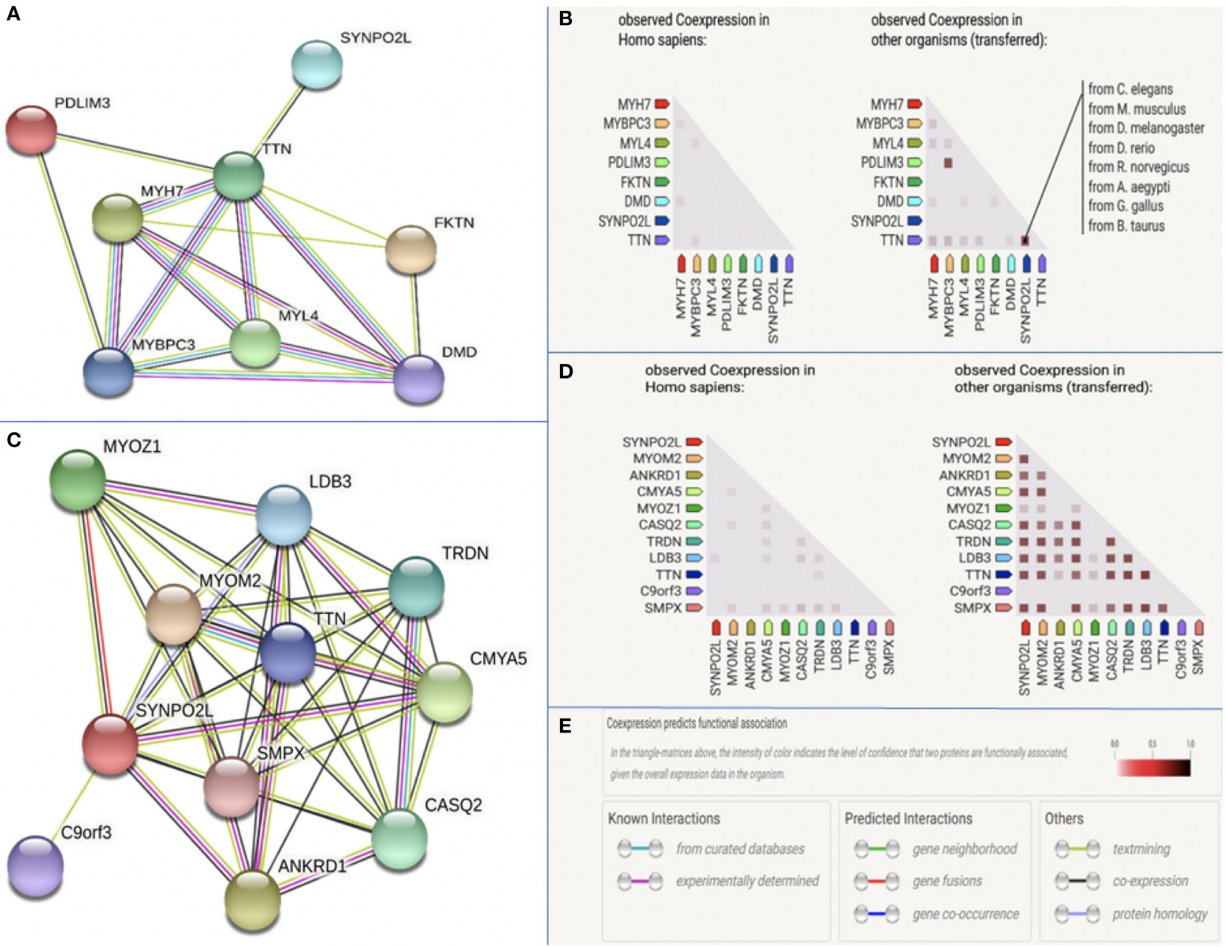
Protein-protein interaction analysis. The figure is modified from data provided by the tool String v11. **(A)** Protein interactions between the product of *SYNPO2L* and seven other structural genes previously associated with AF. **(B)** Co-expression data across Homo Sapiens and other species for the product of *SYNPO2L* and seven other structural genes previously associated with AF. Species in which co-expression has been observed has been highlighted for *SYNPO2L* and *TTN*. **(C)** Protein interactions between the product of *SYNPO2L* and the products of the 10 genes with the highest confidence in interaction prediction. **(D)** Co-expression data across Homo Sapiens and other species for the product of *SYNPO2L* and the products of the 10 genes with the highest confidence in interaction prediction. **(E)** Legend of color codes used in this figures **(A–D)**.

Using the tool String, we examined interactions between the protein product of *SYNPO2L* and those of other structural genes previously associated with AF. The protein product of *SYNPOL2* as well as *TTN* was found to be co-expressed in numerous species as illustrated in Figures 4A–E, indicating a functional association between the protein products of these genes. When searching for interactions between *SYNPO2L* and all genes, we found the *SYNPO2L* to interact with and be co-expressed with the protein products of several other genes. These genes are associated with the structure of sarcomeres, calcium signaling and cardiomyopathies. The proteins interacting with the *SYNPO2L* product are summarized in Supplementary Table 1.

### Variant Deleteriousness Prediction

Using *in silico* analyses we investigated the potential deleteriousness of the rs766868752 variant in the *SYNPO2L* gene. The variant was found to have a CADD score of 35, indicating that it is among the 0.1% most deleterious variants in the human genome.

## Discussion

In a previously performed meta-analysis of GWAS, 134 loci associated with AF were identified in the human genome (4). However, these are all common variants with small effect sizes. In this study we report on the first loss-of-function variant in the FinnGen study significantly associated with atrial fibrillation. This rare variant has a substantial effect size and is found in the *SYNPO2L* gene located on chromosome 10q22. This region has previously been associated with AF as the intronic SNP rs60212594 in *SYNPO2L* has been identified in our GWAS meta-analysis. This common variant was found with an associated OR of 1.12 (p = 6,48 × 10^−27^). Interestingly, a study by Brugada et al. (16) from 1997 identified a family of 26 with AF being inherited in an autosomal dominant pattern. The responsible genetic locus was localized to chromosome 10q22-10q24. Combined with GWAS, this strongly implicate genes in chromosome 10q22 as candidate genes with a potential large significant effect on the risk of AF.

Different studies have suggested both *MYOZ1* (17, 18) and *SYNPO2L* (19, 20) both located on chromosome 10q22, as the likely causal genes driving the association. Studies suggesting *MYOZ1* as the causal gene, have done so primarily based on eQTL studies showing an altered expression of the *MYOZ1* gene in carriers of AF risk alleles identified through GWAS studies. However, expression of the *MYOZ1* gene has been shown to be primarily restricted to skeletal muscle, with very low *MYOZ1* mRNA levels in cardiac muscle tissue (21, 22). Additionally, no variants in the *MYOZ1* gene were found to be significantly enriched in the FinnGen study. These findings suggest *MYOZ1* as an unlikely candidate gene in the locus. Conversely, the findings of this study showing a predicted loss-of-function variant in the *SYNPO2L* gene with an associated OR of 3.5, strongly suggests *SYNPO2L* as the likely causal gene in the locus. This is supported by the colocalization of the AF GWAS signals with the eQTLs of *SYNPO2L* as seen in Figure 2.

### Functions of the SYNPO2L Gene Product

The functions of the *SYNPO2L* gene product were first studied by Beqqali et al. (23), naming the protein product Cytoskeletal Heart-enriched Actin-associated Protein (CHAP). Beqqali et al. identified two different isoforms both expressed in cardiac tissue. The long isoform was named CHAPa (=978 aa) coded by the ENST00000394810.2 transcript, and the short isoform was named CHAPb (=749 aa) coded by the ENST00000372873.4 transcript. The authors furthermore found that the long isoform CHAPa was predominately expressed in adult cardiac tissue, whereas the short isoform, CHAPb, was primarily expressed in embryonic cardiac tissue. Expression of *SYNPO2L* in various tissues can be seen in Supplementary Figure 1 (24). The splice-site variant identified in the FinnGen database is only present in the long isoform coding the CHAPa. Using *in situ* hybrdization in mice, Beqqali et al. determined the subcellular localization of the proteins to the Z-disc of both skeletal and cardiac muscle tissue by co-localization with a-actinin (23). Both the CHAPa and CHAPb isoforms contain a nuclear localization signal (NLS) which was found to be highly conserved in both the mouse and zebrafish orthologs. Accordingly, CHAPb, the embryonic form, was also localized to the nucleus in embryonic mice cardiac tissue.

To further investigate the function of the *SYNPO2L* product, Beqqali et al. knocked down the CHAPa and CHAPb isoforms of the *SYNPO2L* gene product in a zebrafish model using antisense morpholino oligonucleotides (23). The zebrafish kncokdown-models showed a phenotype with highly disorganized sarcomeres and Z-disks, as well as decreased cardiac contractility. α-actinin-2, which was shown to interact with the CHAP proteins, was also displaced resulting in disorganized myofibrils. This suggests that the *SYNPO2L* gene product is highly involved in the structural development and function of the sarcomeres. Due to its nuclear and Z-disc localization, this role could be carried out by the protein acting as both a structural component and also being involved in cellular signaling processes. This is in line with previous findings suggesting other Z-disc proteins to be associated with a-actinin. One example is MLP, which can translocate from the cytoplasm to the nucleus and interact with varius transcription factors (25).

The consequences of splice site mutations on the protein product depend on multiple different factors. These are complex to predict, but can include exon skipping, intron inclusion or activation of different cryptic splice sites (26). If the splice site mutation in intron 1 of *SYNPO2L* leads to a change in the remaining reading frame, the mutation is likely to lead to a pre-mature stop codon followed by non-sense mediated decay (NMD) (27). Therefore, the splice site mutation could dispose to AF either by a lack of protein product or by an altered dysfunctional protein product. The pathogenicity of the splice variant identified in this study is underlined by *in sillico* analysis, predicting the variant to be among the 0.1% variants in the genome most likely to be deleterious. Future analysis of mRNA extracted from cell lines or patients with the splice site variant could help determine how the mutation affects splicing and protein product. Carriers of the common AF risk variant rs60212594 show a decreased expression of *SYNPO2L*, as shown in the eQTL analysis (Figure 3). Expression of *SYNPO2L* in carriers of this common variant was examined as the variant is associated with an increased risk of AF in our GWAS meta-analysis (4). The decreased expression is consistent with the present finding that the rs766868752 splice site variant, possibly leading to mRNA NMD, was strongly associated with AF. Both findings suggest that a decreased amount of *SYNPO2L* gene product pre-disposes to AF.

Genetic variants in other structural cardiac proteins e.g., titin, have previously been shown to be strongly associated with AF (28). *TTN* encodes a giant sarcome protein (titin) expressed in all chambers of the human heart. Ahlberg et al. (28) found sarcomere defects in mutant zebrafish carrying a truncated variant in ttn, the homologs *TTN* gene in zebrafish. This was also the case in the *SYNPO2L* knockdown zebrafish model studied by Beqqali et al. (23). Furthermore, a prolonged PR interval as well as an increased amount of fibrosis was shown in zebra fish models carrying the truncated titin variant (28). Another interesting gene, *MYL4*, is responsible for contractile, electrical and structural integrity of the atrium. Peng et al. found a loss-of function variant to cause early atrial fibrosis leading to atrial arrhytmia and atrial cardiomyopathy (29). In line with this, a mouse model with an overexpression of the *SYNPO2L* gene studied by Van Eldik et al. (30), also displayed both increased interstitial fibrosis and electrical abnormalities. Cardiac fibrosis has been shown to be increased in AF patients (31). The findings of increased cardiac fibrosis and atrial cardiomyopathy, resulting from abnormal sarcomere organization, propose a possible mechanism of how variants in the *SYNPO2L* gene and other structural proteins of the cardiomyocyte pre-dispose to AF.

However, this study also has several limitations. While the association seems biologically plausible as the genetic locus is associated with AF and the variant has been predicted to be deleterious, the splice variant, rs766868752, is found with a very low allele frequency and is almost exclusively found in persons of Finnish heritage. Furthermore, the allele frequency of the variant has been imputed. Although the variant was imputed with a very high confidence in imputation, one must consider the possible risk of inaccuracies in imputation. Finally, the common variant analyzed in this study, rs60212594, while significantly associated with AF, has a quite modest effect on disease risk, and may not be of major clinical significance for individuals carrying this variant. We therefore urge our readers to interpret our results with caution.

## Conclusion and Significance

For many years, AF has been considered an electrical disease, but our understanding of the pathophysiology of AF has improved and the field continues to grow as we explore new associations in AF genetics. Our findings suggest that mutations in the *SYNPO2L* gene encoding the structural protein CHAP pre-dispose to AF. This is primarily based on the loss-of-function splice site variant in the *SYNPO2L* gene that was associated with a significantly increased risk of AF in a Finnish population (*p* = 9.9 × 10^−8^). The loss-of-function variant in the *SYNPO2L* gene is too rare to be of any major clinical significance alone. However, the findings that mutations in another gene involved in the structural arrangement of the sarcomeres pre-dispose to AF, supports the theory that AF is not only an electrophysiological disease. Future studies are needed to investigate if *SYNPO2L* loss-of-function variants in general pre-dispose for AF with a similar effect size. The discovery that atrial fibrosis plays a significant role in AF might explain why existing treatment options of AF are sometimes inadequate and may help identify new directions of AF treatments and risk stratifications in the future. It also underlines the complexity of the disease and suggests that different genes in different geographic regions and populations may pre-dispose for the disease through diverse pathophysiological mechanisms.

## Data Availability Statement

Publicly available datasets were analyzed in this study. This data can be found here: https://www.finngen.fi/en/access_results.

## Ethics Statement

Ethical review and approval was not required for the study on human participants in accordance with the local legislation and institutional requirements. Written informed consent for participation was not required for this study in accordance with the national legislation and the institutional requirements.

## Author Contributions

AC and MO: conceived the idea. AC, MO, and OV: performed the analyses. AC: prepared the original draft. AC, MO, OV, and JA: helped write, edit and review the manuscript for key intellectual content. All authors have read and agreed to the published version of the manuscript.

## Conflict of Interest

The authors declare that the research was conducted in the absence of any commercial or financial relationships that could be construed as a potential conflict of interest.
