# Supplementary material for: Loss-of-Function Variants in the SYNPO2L Gene Are Associated With Atrial Fibrillation
Source: Front Cardiovasc Med. 2021 Mar 9;8:650667. doi: 10.3389/fcvm.2021.650667 (PMC7985167; doi:10.3389/fcvm.2021.650667)
Supplement: Supplementary file 1 [file Image_1.PDF]

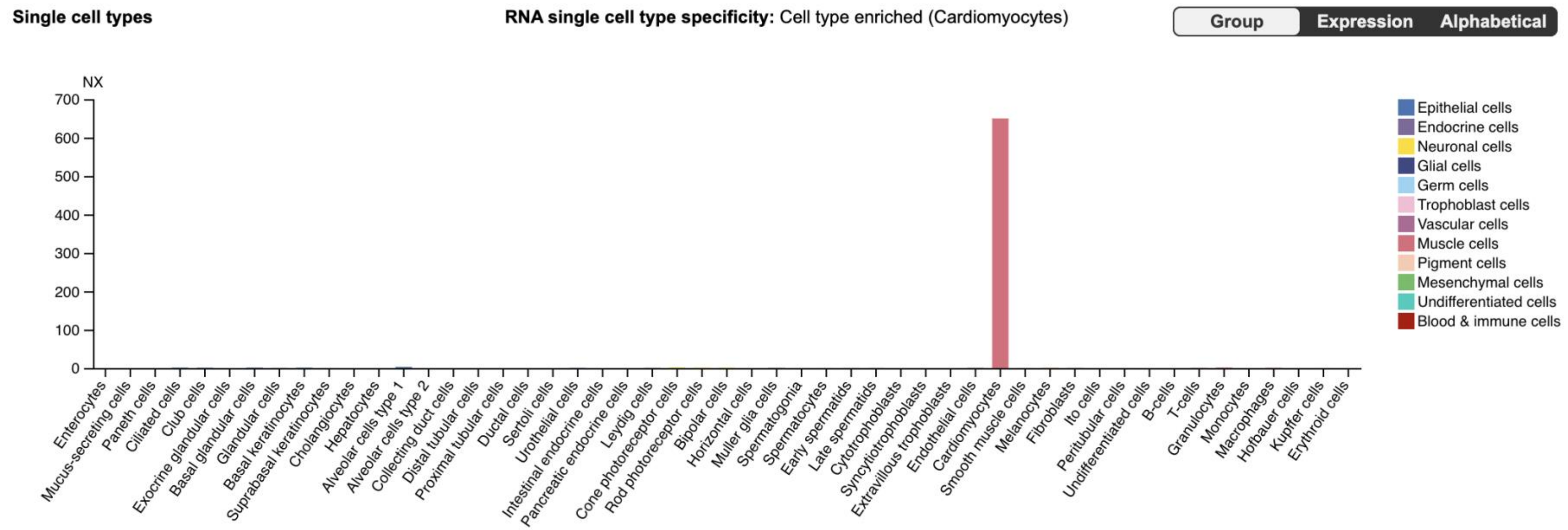

Supplementary figure S1: RNA single cell expression of SYNPO2L. SYNPO2L is almost exclusively expressed in cardiomyocytes. NX = Normalized eXpression. Image credit: Human Protein Atlas. https://www.proteinatlas.org/ENSG00000166317-SYNPO2L/celltype.
