# Supplementary material for: Loss-of-Function Variants in the SYNPO2L Gene Are Associated With Atrial Fibrillation
Source: Front Cardiovasc Med. 2021 Mar 9;8:650667. doi: 10.3389/fcvm.2021.650667 (PMC7985167; doi:10.3389/fcvm.2021.650667)
Supplement: Supplementary file 2 [file Table_1.DOCX]

**Table S1. Proteins interacting with the *SYNPO2L* protein**

| Protein | Gene | Function | Clincal significance | Reference |
| --- | --- | --- | --- | --- |
| Titin | *TTN* | Sarcomere structure | Associated with cardiomyopathy (DCM) and arrhythmia (AF) | PMID: 22335739  PMID: 30333491 |
| Myomesin-2 | *MYOM2* | Stabilizes M-band structure | Candidate gene in the development of HCM | PMID: 33033063 |
| CARP | *ANKRD1* | Transcription factor | Associated with cardiomyopathy (DCM) | PMID: 19525294 |
| Cardiomyopathy-associated protein 5 | *CMYA5* | May serve as anchoring protein and be involved in assembly of Ryanodine receptor clusters | May be associated with cardiomyopathy (HCM) | PMID: 26573135 |
| Myozenin-1 | *MYOZ1* | Involved in structure of sarcomere Z-lines | May be associated with cardiomyopathy (DCM) | PMID: 25634555 |
| calsequestrin 2 | *CASQ2* | Involved in intracellular calcium handling | Associated with arrhythmia (CPVT) | PMID: 28596175 |
| Triadin | *TRDN* | Involved in intracellular calcium handling | May be associated with arrhythmia (CPVT) | PMID: 28596175 |
| LIM domain binding 3 | *LDB3* | Stabilizes sarcomere during contraction | May be involved in familial HCM | PMID: 31333075 |
| Small muscle protein, X-linked | *SMPX* | Involved in protecting sarcolemma membrane from mechanical stress | Not associated with cardiac disease | PMID: 22911656 |
| Chromosome 9 open reading frame 3 | *C9orf3* | May be involved in the renin-angiotensin system | May be associated with AF | PMID: 24763465 |

Abbreviations: AF, Atrial Fibrillation; CPVT, Catecholaminergic Polymorphic Ventricular Tachycardia; DCM, Dilated Cardiomyopathy; HCM, Hypertrophic Cardiomyopathy; PMID, PubMed ID
